# Chishao (*Paeoniae Radix Rubra*) alleviates intra-hepatic cholestasis by modulating NTCP in rats

**DOI:** 10.3389/fphar.2024.1341651

**Published:** 2024-02-01

**Authors:** Xiaoqi Sun, Jing Fang, Nanyuan Fang

**Affiliations:** ^1^ Special Police College, Nanjing Police University, Nanjing, China; ^2^ Department of Infectious Diseases, Affiliated Hospital of Nanjing University of Chinese Medicine, Nanjing, China; ^3^ Department of Chinese internal Medicine, Nanjing University of Chinese Medicine, Nanjing, China

**Keywords:** intra-hepatic cholestasis, Chishao (*Paeoniae Radix Rubra*), ANIT, NTCP, BESP, MRP2

## Abstract

**Background:** Cholestasis is a common pathological manifestation dominated by accumulation of potentially toxic biliary compounds. Na^+^-taurocholate cotransporting polypeptide (NTCP) plays a critical role in protection from cholestasis and can be targeted therapeutically. Chishao (*Paeoniae Radix Rubra*) is a clinically efficacious agent for treating cholestasis, but the underlying mechanism has not been fully clarified.

**Objective:** To evaluate the effects of Chishao on the expression of NTCP in rats with alpha-naphthylisothiocyanate (ANIT)-induced cholestasis.

**Methods:** Chishao extracts were obtained by water decoction. Cholestasis model induced by ANIT in rats were established. Thirty rats were divided into five groups: control group (C), ANIT model group (M), 10 g/kg Chishao group (LD), 20 g/kg Chishao group (MD) and 40 g/kg Chishao group (HD). The levels of serum alanine aminotransferase (ALT), aspartate aminotransferase (AST), total bilirubin (TB), direct bilirubin (DB), alkaline phosphatase (ALP) and total bile acid (TBA) were detected. The mRNA and protein expression of NTCP, multidrug resistance associated protein 2 (MRP2) and bile salt export pump (BSEP) were detected by reverse transcription qPCR and Western blotting respectively. To assess the effects of Chishao on NTCP, MRP2 and BSEP localized at the membrane of hepatocytes, an *in vitro* experiment involving primary hepatocytes was conducted via the utilization of laser scanning confocal microscopy.

**Results:** The extracts of Chishao significantly improved serum ALT, AST, ALP, TB, DB and TBA (*p <* 0.05), especially ALP in the HD group (*p <* 0.01). The histological pathological findings were also reversed in LD, MD and HD groups. The mRNA level of MRP2 was significantly downregulated after treatment with ANIT, whereas it was reversed in MD and HD groups (*p <* 0.05). The mRNA expression of NTCP was significantly downregulated after ANIT treatment, but dramatically upregulated in the HD group. The expressions of BSEP and MRP2 were similar, but that of NTCP decreased after ANIT treatment, which was reversed significantly by Chishao extracts in a dose-dependent manner. The expression of NTCP in hepatocytes from rats increased dose-dependently after Chishao treatment *in vitro*.

**Conclusion:** Chishao extracts can improve the serum and histological performances of intra-hepatic cholestasis caused by ANIT, probably by working on transport proteins in liver cell membranes.

## 1 Introduction

Cholestasis is a pathological manifestation recognized by the disturbance or cessation of bile flow. It is a frequent, severe syndrome induced by various liver diseases, with clinical findings of icterus, pruritus, and fatigue (Wagner and Fickert, 2020). Cholestasis-related hydrophobic bile salts and other bile components chronically accumulating in the hepatocyte and bile capillaries induced activation of inflammatory factors, necrosis, apoptosis and fibrogenesis (Li et al., 2017). The consequence of cholestasis includes injury of hepatocytes and cholangiocytes, which can even progress to the undesirable outcomes including liver fibrosis, cirrhosis, as well as liver failure, hepatobiliary cancers (Bowlus et al., 2019). Hepatic transporters are implicated in the mechanisms of cholestasis (Trauner et al., 1998; Marcelo et al., 2008; Klaassen and Aleksunes, 2010), including sodium-taurocholate cotransporting polypeptide (NTCP, SLC10A1/Slc10a1) (Trauner et al., 2017) and Organic anion transporting polypeptide (OATP), multidrug resistance associated protein (MRP2) and canalicular bile salt export pump (BSEP) (Soroka and Boyer, 2014; Remetic et al., 2022). The first two transporters are found on the basolateral membrane of hepatocytes, where they uptake bile salts, organic anions, and other amphipathic organic solutes from portal blood, while the last two are found on the canalicular membrane of hepatocytes, where they mediate ATP-dependent multi-specific organic anion transport into bile and contribute to bile flow. (Trauner et al., 1998; Boyer, 2007). Animal models of cholestasis have shown that NTCP levels decreased at the transcriptional level (Tanaka et al., 2009), following a protective flowback mechanism to reduce the uptake of potentially hepatotoxic bile acids. As a canalicular efflux transporter highly expressed in hepatocytes, MRP2 (Abcc2) is crucial for the biliary excretion of glutathione and glutathione-conjugated organic anions (Chai et al., 2015). MRP2 expression can be downregulated by alpha-naphthylisothiocyanate (ANIT), ethinylestradiol and other cholestasis-inducing agents or pretreatment with bile duct ligation (Zhang et al., 2016; Yan et al., 2021). ANIT injures epithelial cells of the bile duct and damage bile flow, resulting in intra-hepatic cholestasis. Animal models of cholestasis are usually established with ANIT, following a similar pathological mechanism to that of human cholangiolitic hepatitis (Tanaka et al., 2009).

Chishao is the dried root of *Paeonia lactiflora Pallas* or *Paeonia veitchii Lynch*. Chishao is capable of cooling blood, clearing heat, and invigorating blood circulation in Traditional Chinese medicine (TCM). It is usually prescribed to treat cardiovascular, inflammatory and female reproductive diseases, including hepatitis, blood stagnation manifested as dysmenorrhea, amenorrhea, as well as acute inflammation with redness, swelling and pain from traumas. High-dose Chishao can efficiently downregulate the levels of serum alanine aminotransferase (ALT), aspartate transaminase (AST), total bilirubin (TB) and direct bilirubin (DB) in cholestatic patients (Ma et al., 2014). Animal studies showed that Paeoniflorin, a principal component of Chishao, exerted obvious therapeutic effects on acute cholestatic hepatitis induced by ANIT (Zhao et al., 2013; Zhao et al., 2017), while the underlying mechanisms of the effect of Chishao itself have not been clearly elucidated yet.

In this study, we detected the expressions of hepatocyte membrane transporters in rats with ANIT-induced cholestasis as well as hepatocytes *in vitro*, to clarify the mechanism underlying Chishao protection against cholestasis.

## 2 Materials and methods

### 2.1 Experimental animals and treatment

Thirty male SD rats (200–220 g) were purchased from Shanghai SLAC Laboratory Animal Co., Ltd. (China), Laboratory Animal License No. SCXK (Shanghai) 2012–0002, Animal Qualification Certificate No. 2007000568907. The study was strictly conducted in accordance with the Guidelines for the Care and Use of Laboratory Animals of the Ministry of Science and Technology of China, and the experimental protocol was approved by the Ethics Committee of Animal Experiments of the Nanjing university of Chinese medicine [Laboratory Animal Ethics No. ACU-05 (20130910)]. All the animals were acclimatized for 1 week prior to the experiment and were kept in the same temperature (25°C ± 2°C) and lighting (12:12 h, light: dark cycle), with free access to standard laboratory chow and tap water. The animals were randomly divided into five groups of 6 rats each, including control group (C), ANIT group (M), 10 g/kg Chishao group (LD), 20 g/kg Chishao group (MD) and 40 g/kg Chishao group (HD). All the rats, except for the control group, were intragastrically given 100 mg/kg ANIT (Sigma, United States; dissolved in peanut oil) once on the second week (Chisholm and Dolphin, 1996). Afterwards, the rats in the group of LD, MD, HD were given Chishao extracts seven times (two times per day) at doses of 10, 20, 40 g/kg body weight respectively, while the rats in the group of M and the control group normal saline. All the rats were fasted for 12 h, and sacrificed after an intraperitoneal injection of at least 140 mg/kg sodium pentobarbital in a considerate and painless manner (Scientific Procedures Act 1986). Blood samples were collected and centrifuged at 3000 *g* for 10 min to obtain serum.

### 2.2 Drugs and UPLC-MS

The crude drug of Chishao was provided by Baicaotang TCM Clinic and certified by Prof. Le Wei from Nanjing University of Chinese Medicine. Drug was boiled gently in a 10-fold volume of water for 60 min and filtered, and the filtrate was spray-dried to obtain the extract at a yield of about 10% (by weight) of the original preparation. Then the extract was filtered through a 0.45 μm Fluoropore membrane (Millipore, MA, United States) prior to injection into a UPLC-ESI-MS/MS system. According to the 2015 edition of Chinese Pharmacopoeia, the quality of Chishao can be controlled by detecting paeoniflorin. The quality standard component paeoniflorin was purchased from Chengdu Pufeide Biotechnology Co., Ltd. (China). Paeoniflorin was filtered through a 0.45 μm Fluoropore membrane.

**FIGURE 1:**
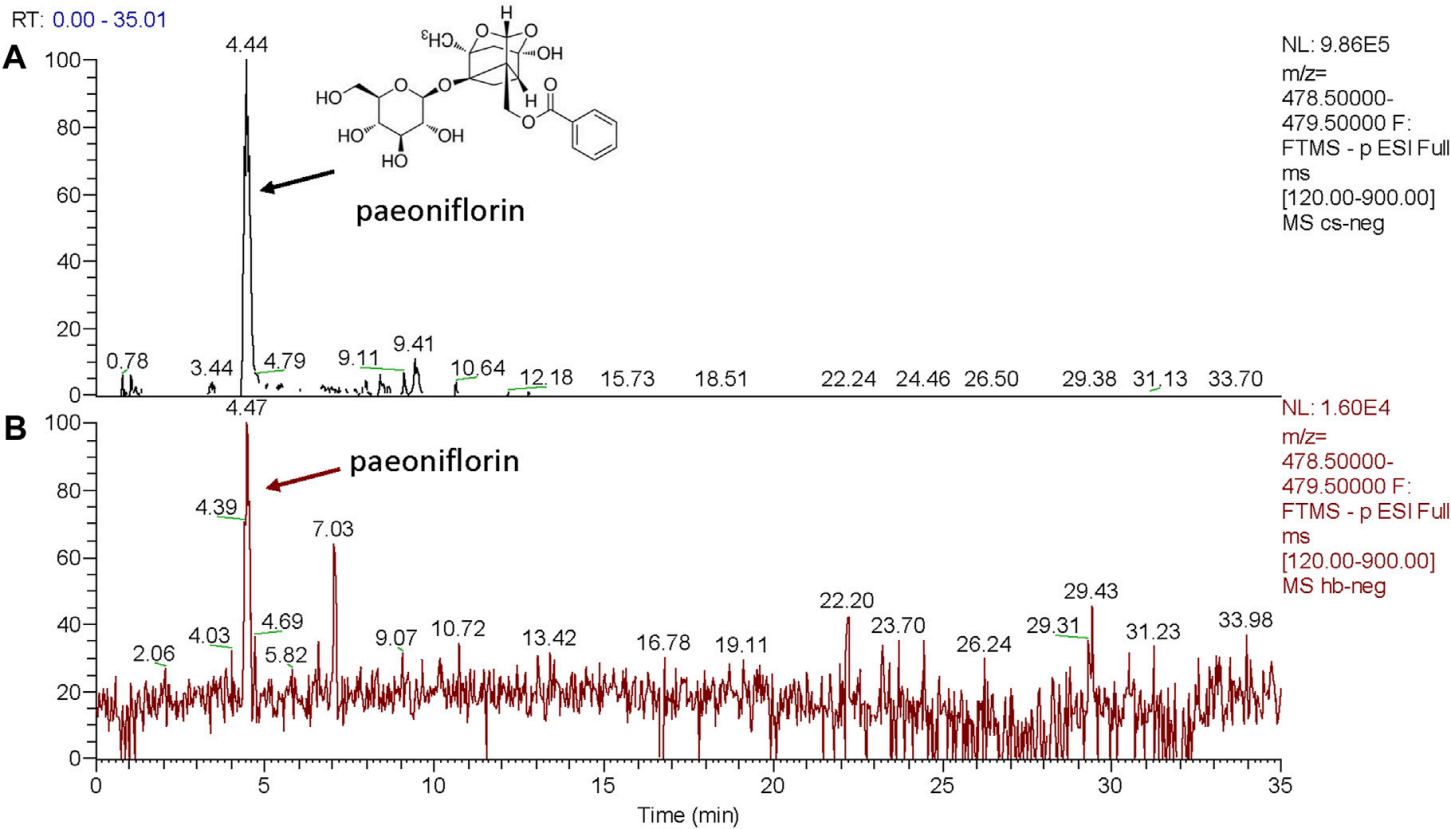
UPLC mass spectrometry analysis of Paeoniflorin standard and Chishao extracts, all compounds were monitored using the ESI-negative ion mode. **(A)** Chemical structures and MS/MS spectra of Paeoniflorin standard; **(B)** MS/MS spectra of Chishao extracts (marked in red).

UPLC analyses were performed by a UPLC-LTQ-Qrbitrap system (Thermo, United States of America) with a TQ detector using an Accucore C18 column (2.1 mm × 150 mm, 0.26 μm) with a binary mobile phase. Solvent A was acetonitrile and B was 0.1% aqueous formic acid solution. The gradient elution at room temperature was 5%–12% (v/v) A at 0–30 min, 40% (v/v) A at 35 min, 40% (v/v) A at 52 min, 70% (v/v) A at 63 min, 85% (v/v) A at 67 min, and 85% (v/v) A at 70 min. The flow rate was 0.35 mL/min and the sample injection volume was 1 μL. LC-MS was performed by a Thermo TQ detector equipped with ESI source. MS was operated in the positive/negative mode, and the data were acquired in the scan mode using a m/z range of 100–1000 (Figure 1).

### 2.3 Serum biochemical analysis

The serum levels of AST, ALT, alkaline phosphatase (ALP), TB, DB and total bile acid (TBA) were assayed by commercially available clinical test kits and an automatic biochemical analyzer (HITACHI 7080, Japan).

### 2.4 Histological assessment

Liver tissue samples from the experimental animals were fixed in 10% buffered formalin, embedded in paraffin, sliced (3–5 μm thick), and counterstained with hematoxylin and eosin. Histological examination for morphological changes were performed in a blinded manner by Hongbao Yan, an independent pathologist from Nanjing Medical University.

### 2.5 Gene expressions

Total RNA from rat liver was harvested in liquid nitrogen, isolated and stored at −70°C before processing. Total RNA was extracted from frozen tissue with RNAiso reagent (Takara, Japan). Reverse transcription was performed using Advantage RT-for-PCR kit (Takara, Japan). Quantitative PCR was performed using SYBR Green PCR Master Mix (Roche) and primers (Table 1) in a 7500 HT thermocycler (Applied Biosystems). The method for calculation of gene expression is 2^‐△Ct^, ΔCt = Ct value of the sample target gene - Ct value of the sample internal reference GAPDH (Schmittgen and Livak, 2008).

**TABLE 1:** Primer sequences for quantitative real-time PCR.

Gene	Sequence (5′–3′)	(GenBank no.)
*Mrp2*	forward: 5′-CCA​ATG​TTT​TGA​ATG​CGG​AG-3′	NM_012833
	reverse: 5′-AGG​ATC​GAT​GAG​GTC​ACC​ATG-3′	
*Bsep*	forward: 5′-TTT​TCC​AGA​GGC​AGC​TAT​CG-3′	U69487
	reverse: 5′-ATG​GCT​GCA​CTC​AAA​GAT​CC-3′	
*Ntcp*	forward: 5′-AGG​CAT​GAT​CAT​CAC​CTT​CC-3′	NM_017047
	reverse: 5′-AAG​TGG​CCC​AAT​GAC​TTC​AG-3′	
*Gapdh*	forward: 5′-AAT​GTA​TCC​GTT​GTG​GAT​CTG​A-3′	NM_017008
	reverse: 5′-GCC​TGC​TTC​ACC​ACC​TTC​T-3′	

### 2.6 Protein expressions

Anti-rat monoclonal NTCP antibody (Abcam), anti-rat polyclonal MRP2 antibody (Abcam), anti-rat polyclonal BSEP antibody (Abcam) and anti-mouse β-actin antibody (Santa Cruz) were used.

Rat liver tissue (100 mg) was homogenized and subsequently lysed in ice-cold lysis buffer with protease inhibitor. The sample was centrifuged at 13000 rpm for 30 min at 4°C, and the supernatant was collected and stored at −70°C for Western blot. Liver protein (50 μg) was separated by 10% SDS-polyacrylamide gel electrophoresis and transferred onto a PVDF membrane. Anti-NTCP, MRP2, BSEP and β-actin antibodies were blocked in 5% fat-free milk. After incubated with HRP-conjugated antibody, the membrane was visualized with chemiluminescence by ECL kit (Thermo, United States of America). Relative expression level was calculated as Intensity Density of target protein/Intensity Density of β-actin in ImageJ software.

### 2.7 *In Vitro* experiment

Hepatocytes were isolated from male SD rats using a two-step collagenase perfusion as described previously (Annaert et al., 2001). The cells were suspended in DMEM supplemented with 10% FBS and 1% penicillin/streptomycin after being centrifuged (50 ×g) for 3 min. Before seeding, the cell viability (>90%) was determined by TrypanR blue exclusion, and hepatocytes were diluted to a final concentration of 1×10^6^ cells/mL. Chishao extracts were administered at doses of 0, 0.625 mg/mL, 1.25 mg/mL, 2.5 mg/mL, 5 mg/mL and 10 mg/mL.

### 2.8 NTCP immunofluorescence

Hepatocytes were cultured on slides until they reached a final concentration of 4×10^5^ cells/mL, after which they were rinsed with PBS and fixed with 4% paraformaldehyde for 15 min. After repeated washing with PBS, the slides were treated with 0.5% Triton X-100 and incubated in PBS containing 1% bovine serum albumin for 30 min at room temperature before being incubated with anti-NTCP antibodies overnight at 4°C. After washing the slides in PBS, they were treated for 1 h with goat anti-rabbit fluorescent secondary antibody (Alexa Fluor647). A Leica TCS SP5 laser confocal microscope was used to image the cells.

### 2.9 Statistical analysis

The data were represented as Mean ± SEM. Differences between group means were calculated by one-way ANOVA. Difference was considered to be statistically significance when *p* ≤ 0.05 or *p* ≤ 0.01. All statistical analyses were performed by R software (version 4.0.5).

## 3 Results

### 3.1 Effects of Chishao on the levels of serum enzymes and components

**FIGURE 2:**
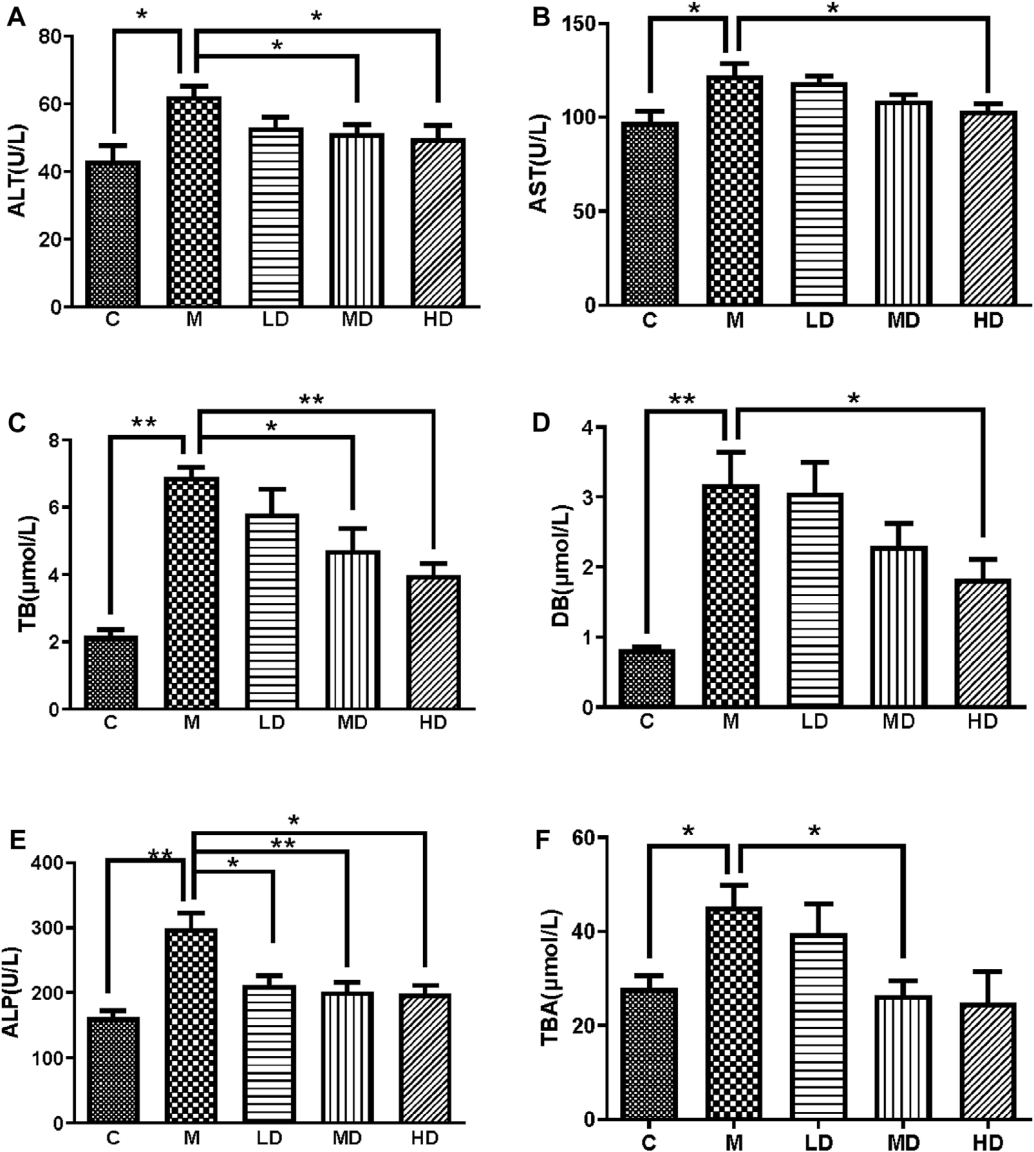
Chishao extracts improved the levels of serum enzymes and components in ANIT-induced cholestatic liver injury in rats **(A–F)**. **(A)** ALT, **(B)** AST, **(C)** TB, **(D)** DB, **(E)** ALT, **(F)** TBA. Values are expressed as mean ± SEM. *p < 0.05, **p < 0.01 vs. ANIT group.

Serum ALT, AST, ALP, TB, DB and TBA levels of the five groups were measured to evaluate liver injury and cholestasis. All the levels were significantly elevated after ANIT treatment compared with those in the control group. Comparing with M group, Chishao extracts significantly and dose-dependently decreased the serum levels of ALT, AST, ALP, TB and DB in LD, MD and HD groups, notably TB and ALP in the HD group. However, TBA was less reversed in the HD group, and the serum levels of liver enzymes in the LD group barely improved. (Figure 2).

### 3.2 Effects of Chishao on histological changes

**FIGURE 3:**
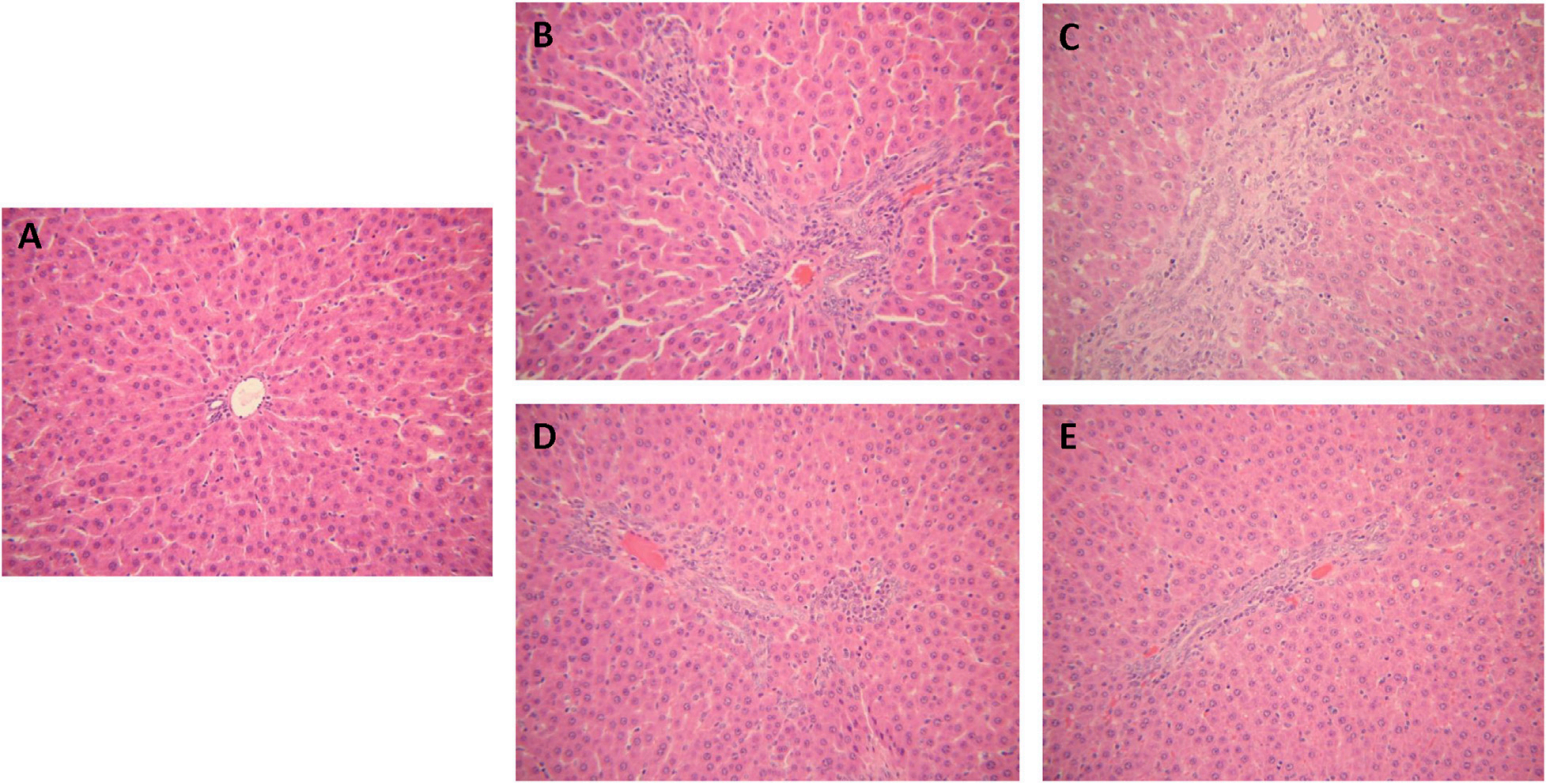
Protection of Chishao against ANIT-induced intra-hepatic cholestasis. Histology of rat liver tissue (H&E). Images were taken under × 200 amplification. **(A)** C group, **(B)** M group, **(C)** LD group, **(D)** MD group, **(E)** HD group.

The hepatic tissues of the control group exhibited a normal cellular structure with distinct hepatic cells and sinusoidal spaces (Figure 3A). In contrast, the slides of liver tissue specimens from the ANIT-treated group presented loss of cellular boundaries, necrotic, degenerative changes and severe interlobular duct epithelial damage with neutrophil infiltration (Figure 3B). The specimens of rats treated with Chishao extracts at the dose of 3 g/kg body weight (HD group) displayed mild necrotic, degenerative changes with less neutrophil infiltration (Figure 3C), which were similar to the control group. With Chishao extracts at the dose of 1.5 g/kg body weight (MD group), the specimens suggested neutrophil infiltration and other histological damages moderately mitigated (Figure 3D). The specimens of rats treated with Chishao extracts at the dose of 0.75 g/kg body weight (LD group) showed the similar severity of damage as that of the ANIT-treated group (Figure 3E).

### 3.3 Effects of Chishao on gene expressions of transporters

**FIGURE 4:**
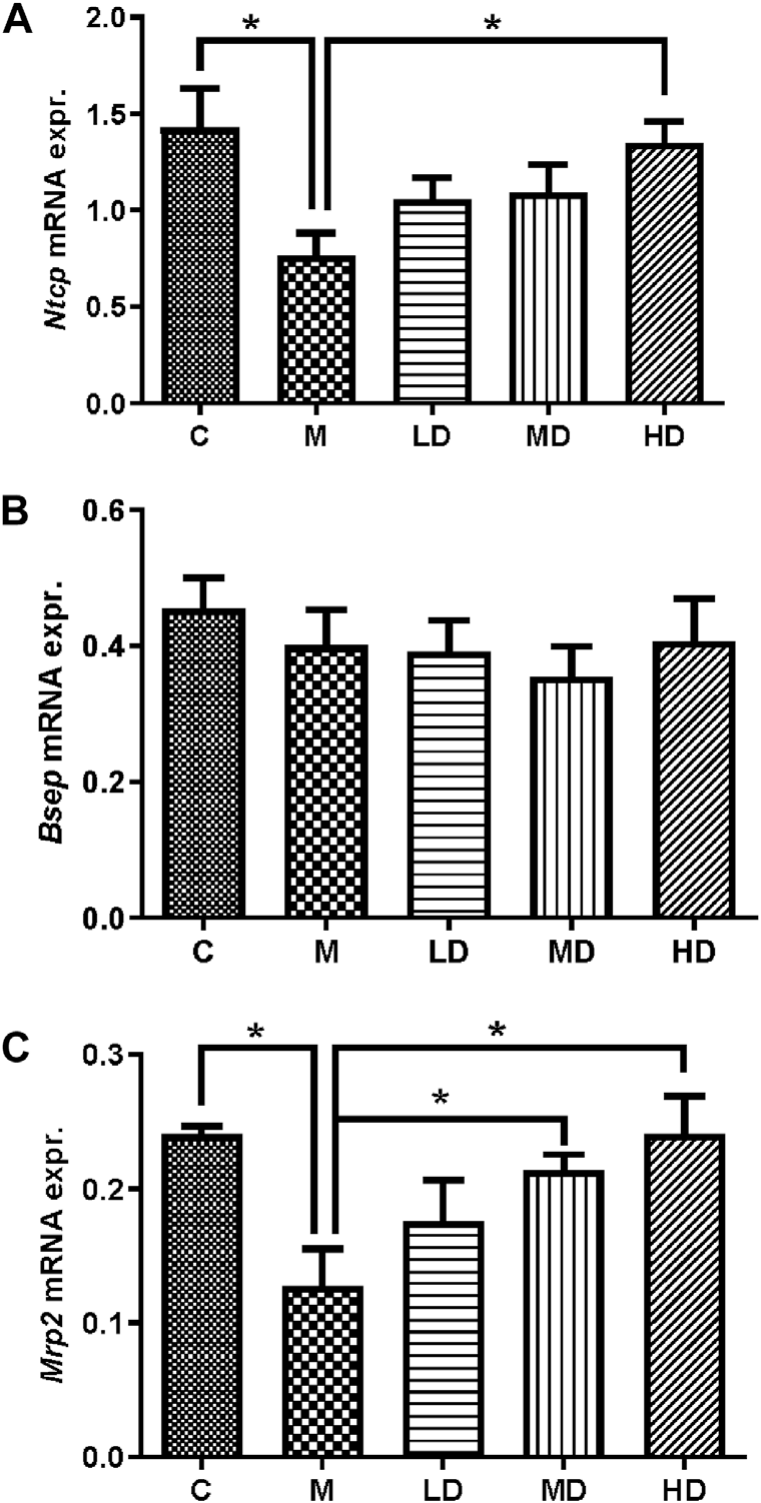
Relative quantification of gene expression by the 2^‐△Ct^ method. The effects of Chishao extracts on the gene expressions of *Ntcp*, *Bsep* and *Mrp2* in ANIT-induced intra-hepatic cholestasis. On day 7 after Chishao fed, rats were sacrificed, the liver tissues were isolated. The mRNA expression of *Ntcp*, *Bsep* and *Mrp2* in left lobe of liver tissues were examined by Quantitative PCR. **(A)** NTCP, **(B)** BSEP, **(C)** MRP2. **p < 0.01 vs. ANIT group (M).

The mRNA expression of liver transporters, including canalicular efflux transporters and basolateral uptake transporters, were investigated. *Mrp2*/*Abcc2* mRNA was considerably downregulated following ANIT administration (*p* = 0.0165), whereas it was reversed in the MD and HD groups (*p* = 0.0467; *p* = 0.0469). *Bsep*/*Abcb11* mRNA did not alter after treatment with ANIT or Chishao extracts (Table 2). Then we turned to *Ntcp*/*Slc10a1* mRNA expression, the results suggested it was substantially decreased after ANIT administration (*p* = 0.0470), while significantly increased in the HD group (*p* = 0.0238) (Table.2; Figure 4).

**TABLE 2:** Relative quantification of transporters gene expressions in ANIT-induced intra-hepatic cholestasis rats (2^‐△Ct^, mean ± SEM).

	C	M	LD	MD	HD
*Mrp2* (*Abcc2*)	0.220 ± 0.031	0.127 ± 0.037 (*p* = 0.0434)	0.176 ± 0.038 (*p* = 0.0467)	0.214 ± 0.014 (*p* = 0.0469)	0.241 ± 0.038 (*p* = 0.0469)
*Bsep* (*Abcb11*)	0.455 ± 0.058	0.400 ± 0.066 (*p* = 0.4730)	0.392 ± 0.058 (*p* = 0.9076)	0.354 ± 0.057 (*p* = 0.5461)	0.407 ± 0.079 (*p* = 0.9391)
*Ntcp* (*Slc10a1*)	1.428 ± 0.239	0.765 ± 0.137 (*p* = 0.0470)	1.058 ± 0.130 (*p* = 0.1425)	1.091 ± 0.177 (*p* = 0.1558)	1.347 ± 0.144 (*p* = 0.0238)

### 3.4 Effects of Chishao extracts on the levels of NTCP, BSEP and MRP2

**FIGURE 5:**
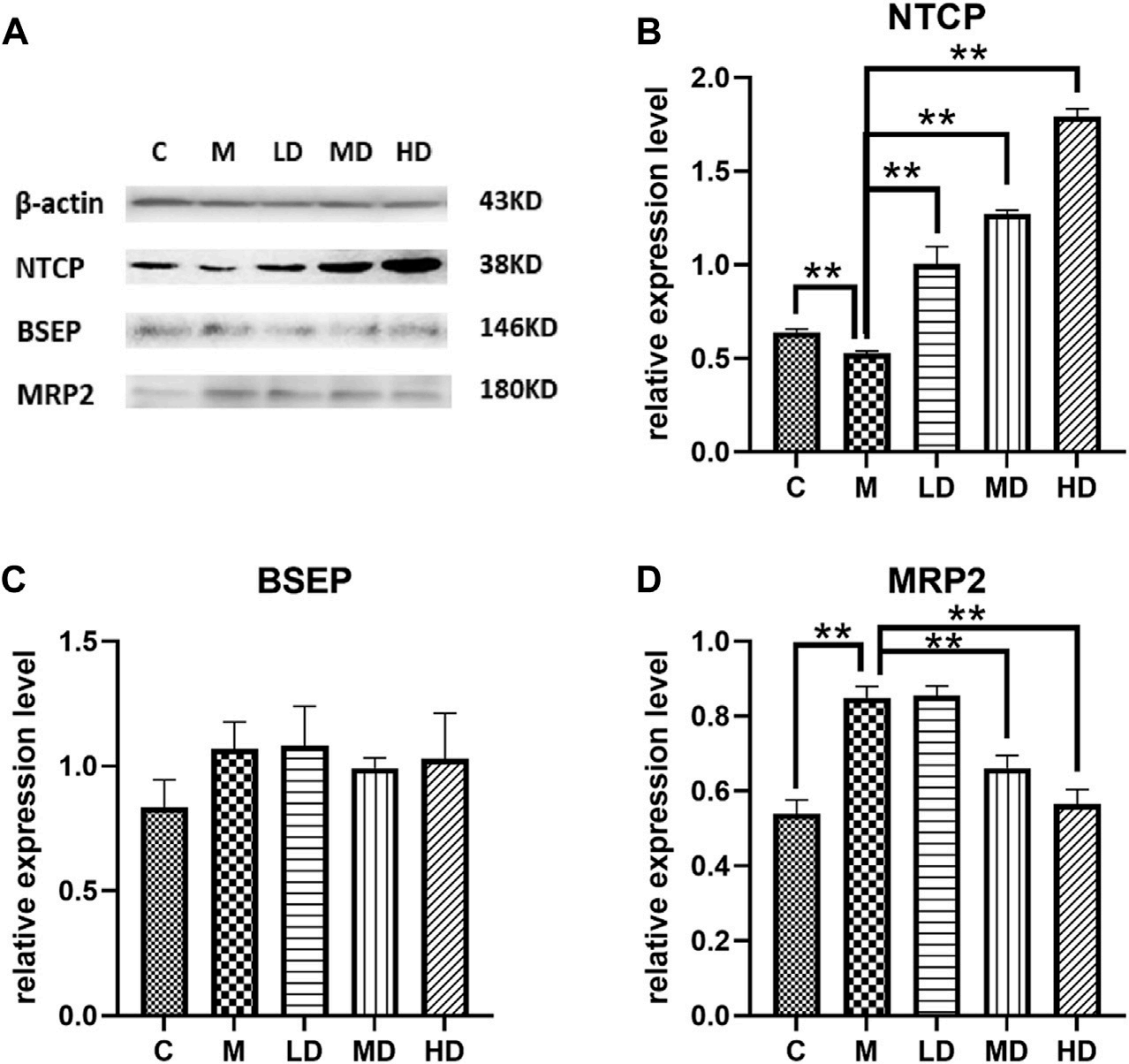
Effects of Chishao extracts on transporter protein levels in rats stimulated by ANIT. **(A)** Representative Western blot analysis of liver NTCP, BSEP and MRP2 after ANIT with or without Chishao extracts. **(B)** Quantitative analysis of NTCP level. **(C)** Quantitative analysis of BSEP level. **(D)** Quantitative analysis of MRP2 level. **p < 0.05 vs. ANIT group.

To further illustrate the effects of Chishao extracts on hepatocellular membrane transporters at the protein level, we identified the levels of NTCP, BSEP, and MRP2. Following ANIT administration, the level of NTCP was decreased, but was restored in a dose-dependent pattern after treatment with Chishao extracts. (Figure 5).

### 3.5 NTCP in rat liver cells by confocal fluorescence microscopy

**FIGURE 6:**
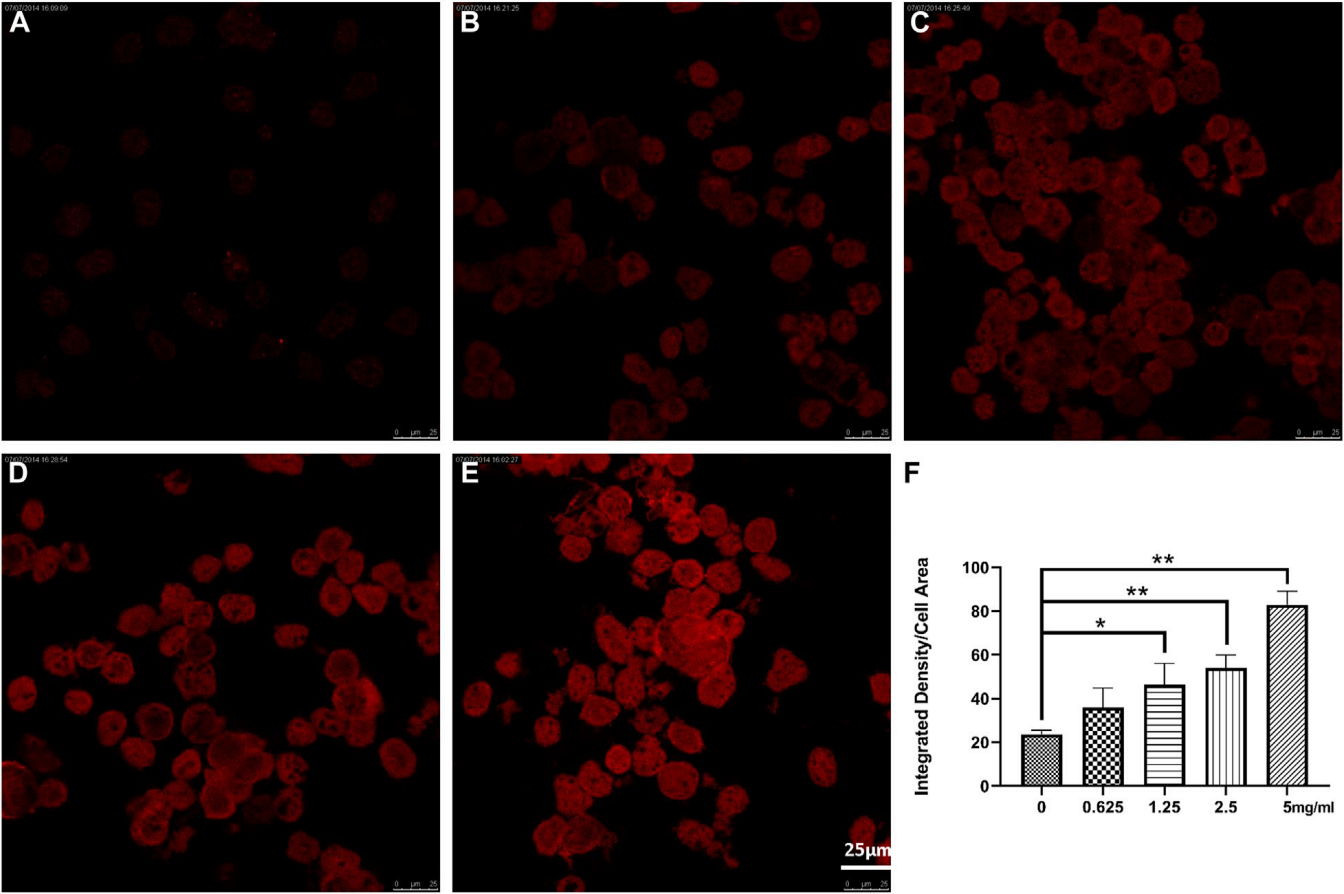
Effects of Chishao extracts on rat hepatocytes *In Vitro* by Confocal fluorescence and quantitative analysis (scale bar = 5 μm). **(A)** 0 mg/mL Chishao. **(B)** 0.625 mg/mL Chishao. **(C)** 1.25 mg/mL Chishao. **(D)** 2.5 mg/mL Chishao. **(E)** 5 mg/mL Chishao. **(F)** Quantitative analysis of rat hepatocytes *In Vitro* with or without Chishao extracts. *p < 0.05, **p < 0.01 vs. 0 mg/mL Chishao group.

**FIGURE 7:**
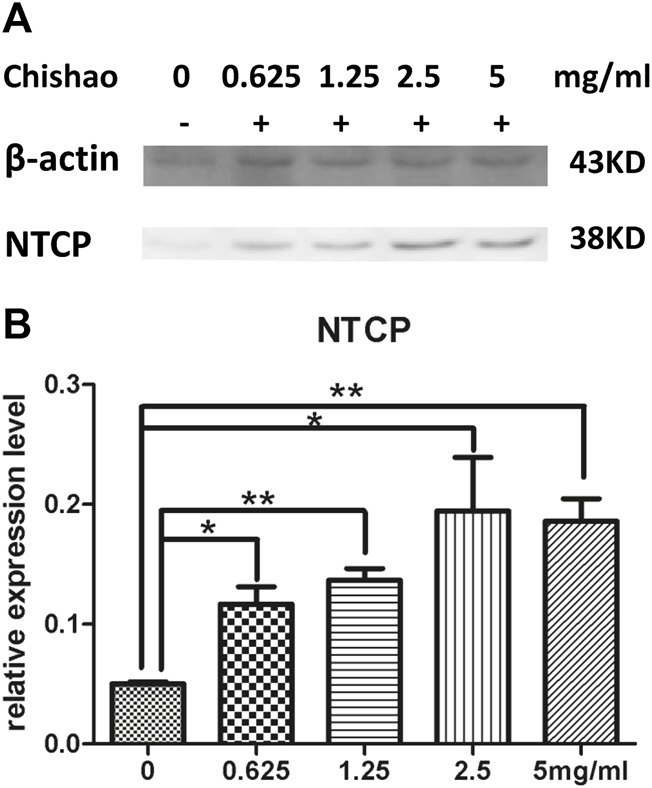
The level of NTCP in rat hepatocytes *In Vitro*. **(A)** NTCP level of hepatocytes were measured using Western blot analysis with or without Chishao extracts. **(B)** Quantitative analysis of NTCP level. *p < 0.05, **p < 0.01 vs. 0 mg/mL Chishao group.

Confocal microscopy showed that NTCP level was upregulated dose-dependently by Chishao extracts in hepatocytes isolated from male SD rats. (Figure 6; Figure 7).

## 4 Discussion

Chishao is one of commonly used herbs in TCM. It is well-documented that cholestasis can be effectively treated by Chishao (Ma et al., 2014), especially at high doses (Wei and Jiang, 2012). From the viewpoint of TCM, the main pathogenesis of cholestasis is heat and stasis in blood, and Chishao can eliminate heat, remove blood stasis and boost microcirculation (Zhao et al., 2023). The ANIT-induced cholestasis in rat has been widely applied to study cholestatic liver disease. The expression of *Mrp2* mRNA was downregulated after treatment with ANIT. Chishao can reverse ANIT-induced liver injury (Huang et al., 2016), previous research reported that high dose of Chishao was in need when cholestasis was aggravated (WC et al., 1983; Lin et al., 2008). The potential mechanism of Chishao protection against cholestasis is little known, especially involving the hepatocellular membrane transporters which dominate in the transport of bile salts and bilirubin.

In this study, ANIT-induced intra-hepatic cholestasis model in rat was established and the therapeutic effects of Chishao extracts was observed, the results suggested that Chishao extracts from water decoction ameliorated the liver function and tissue pathology of the intra-hepatic cholestasis model in rat. Chishao extracts alleviated liver injury by reducing serum levels of ALT, AST, TB, DB, ALP and TBA, which were the markers closely related with intra-hepatic cholestasis. Histological examination revealed that liver tissue in ANIT-treated group showed more severe necrosis, inflammatory cell infiltration, whereas mild damage in Chishao-treated groups. To further explore the protective effects at levels of protein and mRNA, we compared the transcriptions of *Ntcp*, *Mrp2* and *Bsep*. It is found the mRNA of canalicular efflux transporter *Mrp2* was downregulated by ANIT-induced intra-hepatic cholestasis, which was consistent with previous report (Cui et al., 2009). Contrarily, Ding L found that the expression of *Mrp2* mRNA barely changed (Ding et al., 2014). Such inconsistency probably related to the time of drug administration. In our study, the level of MRP2 was increased after ANIT administration, similar to the results of previous research (Cui et al., 2009; Tanaka et al., 2009). Unexpectedly, the level of MRP2 did not decrease after treatment with Chishao extracts at all doses, suggesting that Chishao may not function through MRP2. Another hepatobiliary transporter, BSEP, is one of the members of ATP-binding cassette-dependent transporters. ANIT could decrease the mRNA expression and protein level of liver efflux transporter BSEP in rat (Ou et al., 2016). While in our study, the mRNA and protein expressions of BSEP had no significant variation after ANIT treatment, being consistent with a previous literature (Liu et al., 2003; Ding et al., 2014). NTCP, locating at the hepatocellular sinusoidal membrane, transfers bile acids and organic anions to hepatic cells from the blood (Halilbasic et al., 2013). We validated that *Ntcp* mRNA expression and NTCP level was downregulated upon cholestasis induced by ANIT. When Chishao extracts was given, *Ntcp* mRNA expression and NTCP level was restored dose-dependently. For further exploring the level of NTCP in all Chishao-treated groups, hepatocyte experiment *in vitro* was conducted. It was found that the level of NTCP was augmented by Chishao extracts dose-dependently.

Clinically, high-dose Chishao was able to treat effectively cholestatic patients (Ma et al., 2014). Intra-hepatic cholestasis patient with primary biliary cirrhosis had impaired ability to uptake bile acids and organic anions via NTCP (Zollner et al., 2003). Therefore, the potential mechanism of Chishao extracts may lie in its effectively up-regulating NTCP level in liver cells, which provide evidence for using Chishao in patients with intra-hepatic cholestasis.

In summary, Chishao extracts can improve the serum and histological performances of intra-hepatic cholestasis caused by ANIT, probably by working on transport proteins in liver cell membranes.

## Data Availability

The original contributions presented in the study are included in the article/Supplementary materials, further inquiries can be directed to the corresponding author.
